# Insights Into Medicine Expiry Awareness Among the Population of Rural South India: A Mixed-Methods Approach

**DOI:** 10.7759/cureus.70314

**Published:** 2024-09-27

**Authors:** Rajalakshmi M, Priyanga Datchanamourtty, Prathap Vasigar

**Affiliations:** 1 Community Medicine, Sri Manakula Vinayagar Medical College and Hospital, Puducherry, IND; 2 Orthopaedics, Indira Gandhi Government General Hospital and Post Graduate Institute, Puducherry, IND

**Keywords:** expiry date, health awareness, knowledge, medicine, rural, rural population

## Abstract

Background: The practice of noticing the expiry date of medicines has improved in recent decades. But, still, there are various factors that hinder this practice and make people use medicines that are expired. This study was conducted to assess the knowledge of the expiry date of medicines among the rural population at Thiruvennainallur, in Tamil Nadu, India, and to explore the barriers that hinder the viewing of the expiry date of medicines among the rural population.

Methodology: The study involved both the quantitative phase and the qualitative phase. In the quantitative phase, consenting participants who were residing in the service areas of our Rural Health Training Center (RHTC) at Thiruvennainallur were recruited as study participants by systematic random sampling technique. For the qualitative phase, we conducted in-depth interviews with the study participants using the purposive sampling method.

Results: Out of 182 participants, the number of females (92, 50.5%) was more when compared to males. The majority of the participants were married (152, 85.7%). Around 84 (46.2%) of participants had completed secondary education, and 80 (44%) belonged to Class IV socioeconomic status. About 80.2% purchased medicine using a prescription. The majority of the participants had the awareness that manufactured medicines can be used only for a limited period of time (70.3%) and awareness that expired medicines can cause harm (155, 85.2%). Tablets (159, 87.4%) were the most common form of medicine used. The majority, around 63 (34.6%) of the respondents, considered illiteracy as a reason for not checking the expiration date of medicines.

Conclusion: Lack of knowledge regarding the expiry date of medicine and not considering it as necessary were the major reasons for not noticing the expiry date of medicine by the majority of the respondents. Creating awareness about the ill effects of expired medicines would improve the practice of noticing the expiry date of the medicine.

## Introduction

Many diseases and medical issues can be diagnosed, prevented, and treated with the use of medicines [1]. More than half of all prescription drugs are given out incorrectly, with only 50% of patients taking their prescribed medicines as directed [2,3]. In underdeveloped nations, medication adherence is much lower [4]. In the end, pharmaceutical waste is accumulating in households and finding its way into landfills, sewage systems, streams, and water sources. Medicines are lifesaving and should be used appropriately. If they are consumed after their shelf life, it can have a detrimental effect on one’s health. The shelf life is based on the expiry date of the medicine. The expiry date of a drug is defined as “the date placed on the container of a drug product designating the time during which a batch of a product is expected to remain within the approved shelf-life specification if stored under defined conditions and after which it may not be used” [5].

Understanding the expiration date, potential side effects and interactions of medications is crucial. Despite the easy accessibility to drugs nowadays, it is essential for people to gain knowledge regarding the expiry date. There is still a significant gap in people's understanding of medicine expiry dates. The harmful effects of expired medicines are not widely known, especially in rural settings. Health education plays a crucial role in creating awareness regarding the expiration date of medicine and also in the storage of unused medicines in households. Medical experts, including doctors and pharmaceutical experts, should educate the public on the dangers associated with using expired medicines.

According to the World Health Organization (WHO), every pharmaceutical product should have a defined package pamphlet that gives all the essential information regarding the indications of medicines, adverse effects, interactions, and the date of expiration. The United States Food and Drug Administration (FDA) has been obligated to mention expiration dates on every pharmaceutical product by pharmaceutical manufacturers since 1979 [6]. Apart from this, there is also the practice of using medicines without professional consultation, and over-the-counter medications are also in practice.

The WHO reports that when patients fail to adhere to their prescribed medication regimens, their friends and family members who are familiar with these medications may employ them in times of necessity without seeking professional advice. Medicines should not be consumed beyond the given expiry date. People frequently store leftover and unused medications at home for later use. Misuse and abuse can also occur because of these leftover medicines. Leftover medicines should be handled with care to avoid accidents and risks, particularly for vulnerable groups such as children, the elderly, and pregnant women [7]. Even these unused or expired medicines can pose a threat to the surrounding environment or the water system if not disposed of properly.

The habit of checking the expiry date of medicines has been limited, and various factors hinder people from doing so. Common reasons for leftover medicines include improved health conditions or symptomatic relief, changes in prescriptions or regimens made by prescribers, saving medications for future use, and intolerable side effects [8]. Additional factors contributing to leftover medicines at home include language barriers, the color of the print, font size, and the placement of the expiry date.

While many studies worldwide have explored the barriers to noticing the expiry date of medicines, solutions to these issues have not been adequately addressed. With this background, we aimed to assess the knowledge of the expiry date of medicines and the barriers that hinder people from noticing it. This study was conducted to evaluate the knowledge of the expiry date of medicines among the rural population of Thiruvennainallur in Tamil Nadu, India, and to explore the barriers that prevent them from checking the expiry date of medicines.

## Materials and methods

The study was conducted in a rural area of Thiruvennainallur, serviced by a Rural Health Training Centre (RHTC). The choice of this setting is crucial because it represents a typical rural environment where awareness about medicine expiry dates might be limited. The specific focus on this area allowed the researchers to gain insights into the practices and knowledge levels of a population that may be more vulnerable to the misuse of expired medications.

Study design

The study was conducted as a community-based explanatory mixed-methods study. The study employed a mixed-methods approach, which involved both quantitative and qualitative phases. This approach is beneficial as it allows for a comprehensive analysis of the issue at hand, combining numerical data with in-depth personal insights to understand the phenomenon fully.

The quantitative part of the study consisted of a cross-sectional survey in which a pretested questionnaire was used to interview participants to assess their knowledge and practices related to noticing the expiry date of medicines. The qualitative part involved in-depth interviews to explore the barriers that hinder the viewing of the expiry date of medicines among the rural population. The study participants included individuals over 18 years of age from the field practice area of our RHTC.

Study duration

The study was conducted for three months after getting Institutional Ethical Committee (IEC) clearance. The present study was cleared by the Research Committee and the IEC (Human Studies) of Sri Manakula Vinayagar Medical College and Hospital (SMVMCH), Puducherry, India (IEC no. 132/2024). 

Sample size

Considering the prevalence of knowledge of the expiry date of medicine as 50% and precision as 5%, the sample size was calculated to be 166. Considering the 10% non-response rate, the required sample size for the study was 182 participants [6]. A qualitative interview was conducted among 20 vocal participants to the point of saturation until no new information was further generated. 

Quantitative phase

Sampling Method

The participants were selected through systematic random sampling from the population residing within the service areas of the RHTC. This method ensures that every member of the population has an equal chance of being selected, thereby reducing selection bias and enhancing the generalizability of the findings.

Data Collection

After obtaining the informed consent, the study participants were assessed through a pre-tested questionnaire. The questionnaire included details on sociodemographic variables and knowledge and practice of noticing the expiry date of medicines. The study gathered data on various aspects, such as demographic information, awareness, and practices related to checking the expiry dates of medicines. The use of a structured questionnaire likely facilitated the collection of consistent and comparable data across participants.

Qualitative phase

Sampling Method

For this phase, purposive sampling was employed to select participants for in-depth interviews. Purposive sampling is appropriate when the researcher wants to gather detailed information from specific individuals who have particular knowledge or experience relevant to the research question.

Data Collection

The in-depth interviews provided richer qualitative data, offering insights into the barriers and facilitators to checking expiry dates that are not easily captured in quantitative surveys. This method helps in understanding the reasons behind the observed behaviors and attitudes. The interview was conducted by the investigator trained in qualitative research using an interview guide at the participants' convenient time and venue. One-on-one interviews were conducted in the language comfortable to the participants. After obtaining consent, all the interviews were audio-recorded, and a transcript was prepared. The manual content analysis of the transcripts was done.

Data analysis

Quantitative Analysis

Quantitative data was entered, and analysis was done using the EpiInfo software, version 7.1.5.0, developed by the CDC (Atlanta, GA) and the WHO (Geneva, Switzerland). Continuous variables like age were summarized using mean (SD). Categorical variables like sex and knowledge of the expiry date of medicine were summarized using percentages. The chi-square test was applied to proportions to test the level of significance. The level of significance is fixed at 0.05 with a confidence interval of 95%.

Qualitative Analysis

The data were manually coded, and analysis was done by the principal investigator. To increase the validity of the results, the investigator and a trained faculty in basic research together carried out the analysis. Any discrepancy between the two was resolved by mutual discussion.

## Results

The study included 182 participants, with a nearly equal gender distribution: 92 females (50.5%) and 90 males (49.5%). All participants (100%) identified as Hindu. In terms of marital status, the majority were married, accounting for 156 participants (85.7%), while 26 participants (14.3%) were unmarried. Regarding educational qualifications, 84 participants (46.2%) had completed secondary school, and 23 participants (12.6%) were illiterate. Employment status varied, with 62 participants (34.1%) engaged in other forms of business such as owning a business, working in government jobs, or factories or shops; 53 participants (29.1%) were homemakers, 51 participants (28%) were daily wagers, and 16 participants (8.8%) were unemployed. Socioeconomically, nearly half of the participants (80, 44%), were classified as belonging to the lower middle class according to the Modified B.G. Prasad scale 2024 (Table 1) [9].

**Table 1:** Sociodemographic profile of the study participants (n=182) * Defined as per the Modified BG Prasad Scale 2024 [9]

Variables	Frequency	Percentage (%)
Age (Mean ± SD)	28.21 ± 11.15 years	
Gender		
Male	90	49.5
Female	92	50.5
Religion		
Hindu	182	100
Marital status		
Unmarried	26	14.3
Married	156	85.7
Level of education		
No formal education (illiterate)	23	12.6
Elementary school (Grades 1-8)	49	26.9
Secondary (Grades 9-12)	84	46.2
College and above	26	14.3
Occupation		
Unemployed	16	8.8
Daily wager	51	28
Homemaker	53	29.1
Others (own business, government job, working in factory or shops)	62	34.1
Socioeconomic status *		
Class I	6	3.3
Class II	18	9.9
Class III	58	31.9
Class IV	80	44
Class V	20	11

As per Table 2, 128 of the participants (70.3%) had awareness regarding the expiry date of medicine. Only 71 (39%) study participants had awareness that the expiry date on the bottle differs after bottle opening. While 70.3% of participants had the awareness that the damaged part of the expired drug represents 100%. Almost 159 (87.4%) of the study participants commonly used medication in the tablet form. Nearly half of the study participants (99, 54.4%) suggested creating awareness about the expiration date of medicine to control the hazardous effects of unused and expired medicine. About 123 (67.6%) of study participants recommended that physicians should create public awareness about the proper disposal of unused and expired medicines.

**Table 2:** Participants' awareness regarding the expiration date of medicines (n = 182)

Variables	Frequency	Percentage (%)
Knowledge of what is the expiry date of medicines	128	70.3
Awareness regarding manufactured medicines can be used only for a limited period of time	128	70.3
Awareness of the specific time period mentioned on the packs of the medicines as expiry dates	126	69.2
The expiry date on the bottle differs after the bottle's opening	71	39
Awareness regarding fact that the usage of expired medicine can cause harm	155	85.2
Regarding the provision of advice on the expiry date of medicines by doctors and healthcare professionals	53	29.1
Awareness of what the damaged part of the expired drug represents		
100%	128	70.3
90%	28	15.4
Do not know	26	14.3
Common forms of medicines used		
Tablet	159	87.4
Tablet and capsule	10	5.5
Tablet and syrup	13	7.1
Awareness related to control of the hazardous effects of unused and expired medicines		
Providing proper guidance to the consumer	41	22.5
Creating awareness about the expiry date of medicine	99	54.4
Proper disposal of expired medicine	22	12.1
Providing adequate information	8	4.4
Providing guidance and creating awareness	7	3.8
Creating awareness and providing adequate information	5	2.7
Awareness regarding the role of creating public awareness about proper disposal of unused and expired medicines		
Mass media	39	21.4
Physician	123	67.6
Pharmacy	15	8.2
Mass media and physicians	5	2.7

As shown in Table 3, more than half of the study participants (109, 59.9%) had the habit of checking the expiration date of medicines. Around 63 (34.6%) study participants said that illiteracy was a major reason for not noticing the expiry date, followed by 46 (25.3%) of the study participants who did not consider it necessary to check the expiry date. About 90 (49.5%) study participants periodically checked the expiry date of stored medicines at the time of usage, and 36.3% did not check the expiry date of stored medicines.

**Table 3:** Practice of noticing the expiration dates of medicines (n=182)

Variables	Frequency	Percentage (%)
Practice of checking the expiration date of your medication		
Yes	109	59.9
No	73	40.1
Practice of checking the expiration date of medication while purchasing		
Yes	83	45.6
No	99	54.4
Practice of checking the expiration date on the labels/leaflets of your medicine		
Yes	88	48.4
No	94	51.6
Practice of checking the expiration date before using the medicines		
Yes	103	56.6
No	79	43.4
Presence of any unused drugs in your home		
Yes	67	36.8
No	115	63.2
Reasons for not checking the expiration dates while purchasing medicines		
Laziness	29	15.9
Lack of time	29	15.9
Do not consider it necessary	46	25.3
Pharmacist already checked	15	8.2
Illiteracy	63	34.6
Period after which the expiry date of stored medicines checked		
At the time of usage	90	49.5
Every six months	11	6
Monthly	15	8.2
Do not do so	66	36.3

The results from Table 4 show the association between the sociodemographic profile of the study participants and not noticing the expiry date of medicine. Variables like gender, marital status, level of education, occupation, and monthly income were found to be statistically significant since the p-value < 0.05. 

**Table 4:** Association between the sociodemographic profile of the study participants and not noticing the expiry date of medicines (n = 182) p-value<0.05 significant

Variables	Not noticing the expiry date of medicines	Chi-square value (X^2^)	p-value
Gender			
Male	42	4.29	0.038
Female	57		
Marital status			
Married	8	6.83	0.009
Unmarried	91		
Level of education			
No formal education (illiterate)	23	39.8	<0.001
Elementary school (Grades 1-8)	30		
Secondary (Grades 9-12)	43		
College and above	3		
Occupation			
Unemployed	8	17.6	<0.001
Daily wage	39		
Homemaker	29		
Others	23		
Monthly income (in rupees)			
8,220 and above	2	12.5	0.014
4,110-8,219	8		
2,465-4,109	23		
1,230-2,464	52		
Less than 1,230	14		

Table 5 shows the barriers and solutions to viewing the expiry date of medicines. The barriers and their solutions were classified into four categories, such as knowledge, label factor, language, and miscellaneous. The majority of the study participants found that lack of knowledge, smaller font size, and then language were the major barriers to viewing the expiry date of medicine. The major solutions suggested by the study participants were individualized counseling sessions with people regarding expiry dates, increasing the size of the font, modifying the label color to indicate the expiry date distinctly, and making the local language in the label easy to read. 

**Table 5:** Barriers to viewing the expiry date of medicines and solutions suggested by the participants (n = 20) (): number of people

Barriers	Solutions
Knowledge	
Lack of awareness regarding the expiry date of medicine and do not consider it as necessary (7)	Create awareness of expiry date of medicine (7)
Label factors	
The size of the font is small (4)	Increase the size of the font (4)
Monotonous label/difficulty in differentiating the expiry date from other words (2)	Modify the label’s color to indicate the expiry date distinctly (2)
The expiry date is printed in the part below or unrecognizable area (1)	The expiry date should be mentioned close to the brand name (1)
No indication mark near the expiry date (1)	The caution symbol should be present near the expiry date (1)
Language	
Unable to understand the language in the label (2)	If the label is in local language, it will be easy to read (2)
Employing medications solely in emergencies (1)	Individualized counseling sessions for people and emphasizing proper medication use (1)
Miscellaneous	
People who are alone (1)	Counseling for behavior change (2)
Habitual oversight of medicine expiry (1)	

## Discussion

The present study is to assess the knowledge of the expiry date of medicines among the rural population at Thiruvennainallur. In our study from the quantitative part, we found that nearly three-fourths (70.3%) of study participants knew about the expiry date of medicines. Similar findings on knowledge regarding the expiry date of medicines were found in studies conducted by Gidey et al. (60.7%), and Eltaib et al. Around 71% knew about the expiry date of medicine [6, 8]. A study conducted in Saudi Arabia by Wajid et al. showed that the most commonly used drugs were nonsteroidal anti-inflammatory drugs (80.7%) and antibiotics (48.7%) [10]. While in our study, the majority (87.4%) of the study participants commonly used medicines in tablet forms, followed by 7.1% of participants who consumed tablet and syrup forms. While tablet and capsule forms were consumed by 5.5% of the study participants. Eltaib et al. study represented about 53% of the participants were aware that the expiry date on the bottle differs after bottle opening. One-fifth (24%) of participants did not check the expiry date while purchasing medications [6]. A study by Jha et al. showed that 59.5% of participants had always checked the expiration date of medicines [7]. Similarly, in our study, 59.9% of participants had practiced checking the expiration date of the medicine. While 39% of study participants had an awareness that the expiry date on the bottle differs after the bottle opening.

As per the qualitative results, similar findings from the studies conducted by Eltaib et al., Jha et al., Gidey et al., Wajid et al., and Woldeyohanins et al. found that lack of awareness was a hindrance in seeing the expiry date of medicines [6-8, 10-11]. Many other studies also support the fact that improper labeling is a hindrance while viewing the expiry date of medicine [12-18].

As per the study conducted by Gidey et al., more than half of the study participants (67.4%) suggested that physicians had a responsibility to create public awareness about the proper disposal of unused and expired medicines [8]. From a study by Wajid et al., the prevalence of unused medicine was 89.3%. Of the participants, 55.2% checked the expiration date of the medicine before they purchased it. Around 65% of them kept the medicine until it expired [10]. As per our study results, 36.8% of study participants had unused medicines at home. Likewise, in the present study, 67.6% of participants said that physicians are responsible for creating public awareness about the proper disposal of unused and expired medicines.

A study by Woldeyohanins et al. revealed that 55.9% of participants had unused medicines at home. Half of the participants (53.5%) strongly agreed that unused medicines can cause potential risk at home, and 69.1% of study participants strongly agreed that children are more vulnerable to the potential risk of unused medicines at home [11]. From our study findings, it was found that 85.2% of participants had an awareness that the usage of expired medicines can cause harm. In a study in Pakistan by Shoaib et al., 87% of the respondents had unused medicines in their homes and 55% of the respondents never checked the expiration date before using the medications. About 27% of the respondents' medicines were kept at home even after being expired [12]. In our study, 40.1% of respondents had not checked the expiration date of the medicine.

Strengths

We have conducted an explanatory mixed-method study to find the prevalence as well as hindering factors in the practice of noticing the expiry date of medicines. The sequential use of quantitative and qualitative phases helped us to improve the study's rigor. As we used this type of methodological triangulation in our study, we were able to obtain comprehensive data, which enhanced the understanding of our study phenomenon and provided confirmation of our findings, thus increasing the validity of our study.

Limitations

Being a cross-sectional study, it could not establish a temporal relationship between variables, limiting the ability to draw conclusions about causality. Additionally, the study was conducted in a single rural area, which may affect the generalizability of the findings to other populations or settings. 

The study recommends conducting longitudinal studies to better understand the causal relationships between awareness and practices related to medicine expiry dates. Expanding the research to include diverse geographic areas would also help in generalizing the findings. Furthermore, there is a need for targeted educational interventions to raise awareness about the importance of checking medicine expiry dates, especially in rural areas where literacy levels may be lower. Collaborating with healthcare providers and leveraging mass media could enhance the effectiveness of these interventions.

## Conclusions

Nearly three-fourths of the study participants had awareness regarding the expiration date of medicines. While half of the study participants had the practice of viewing the expiration date of the medicine, the lack of knowledge regarding the expiry date of medicines and not considering it necessary were the major reasons given by the majority of the respondents. Creating awareness about the ill effects of expired medicines would improve the practice of noticing the expiry date of medicines. Increasing public awareness about the harmful effects of using expired medicines and implementing educational interventions can potentially improve the practice of checking expiry dates. Such actions are crucial in ensuring safe medication practices, particularly in rural areas where literacy and access to healthcare information may be limited. ​
